# Cutaneous Poison

**Published:** 1851-01

**Authors:** 


### ARTICLE XII.

### *Case of Annual Return of Cutaneous Poison.*

A. L——, a young lady, now 22 years of age, was exposed in the summer of 1838, to the acrid vapors of the *rhus toxicodendron,* or poison oak. A smarting sensation in the face, occurring after washing with cold water, followed by redness and swelling, induced the belief that she was poisoned by the plant, which she had met with in her walks; the subsequent vesication, and desquamation of the cuticle, confirmed her suspicion. The treatment was palliative, and with the decline of the symptoms, and return of usual health, the affection was almost forgotten. In the year 1839, and about the same time in the year, the disease again returned, and has continued to do so every year since, except the last. She was then on a visit to Virginia; and while there wrote home that it would mar her enjoyment very much to be confined, as usual, with this disease, and desired me to prescribe for its prevention. I did so reluctantly. Alterative doses of iodide of potassium were recommended to be taken daily, for several weeks before the expected attack. She complied with my instructions and had no return of her annual sickness. Her general health, however, was not so good, during the remainder of the year, as it had formerly been. She suffered a good deal from headache, fugitive pains in different parts of the body, irregular appetite, and deficient menstruation. This summer the disease has again returned, and she is now convalescing from a mild attack, which has been mostly confined to the lower extremities. She looks well, and probably will be better than she has been for a year past.—*N. J. Med. Rep.*

